# Influence of Overnight Orthokeratology on Corneal Surface Shape and Optical Quality

**DOI:** 10.1155/2017/3279821

**Published:** 2017-10-01

**Authors:** Yuan Sun, Lin Wang, Jing Gao, Mei Yang, Qi Zhao

**Affiliations:** Department of Ophthalmology, The Second Hospital of Dalian Medical University, Dalian, Liaoning 116027, China

## Abstract

**Purpose:**

To investigate the changes of corneal surface shape and optical quality during orthokeratology.

**Methods:**

49 eyes of 26 patients (10.63 ± 2.02 years old) who underwent overnight orthokeratology for myopia were prospectively examined. The corneal surface shape parameters, including surface regularity index (SRI) and surface asymmetry index (SAI), were attained with an OPD-III SCAN. The higher-order aberrations and higher-order Strehl ratios were calculated under a 3 mm pupil diameter before orthokeratology, 1 month, 3 months, and 6 months after orthokeratology. A *P* value of less than 0.05 was statistically significant.

**Results:**

Months after orthokeratology, SRI and SAI were both showing a significant increase in comparison with those before orthokeratology (*P* < 0.001). After orthokeratology, for a 3 mm pupil, the higher-order Strehl ratio presented a reduction of 0.217 *μ*m (*P* < 0.001), and the higher-order aberration root mean square (HOA RMS) showed a mean increase of 0.100 *μ*m (*P* < 0.001). There were significant increases in spherical aberration (*P* < 0.001) and coma (*P* = 0.044) after orthokeratology. Trefoil showed a slight reduction at month 6 after orthokeratology, but there was no statistical significance (*P* = 0.722).

**Conclusion:**

Overnight orthokeratology for a correction of myopia resulted in a significant improvement in refractive error but increased corneal irregularity and ocular higher-order aberrations, especially in spherical aberration.

## 1. Introduction

Myopia, the most common eye disorder, is caused by the increase of axial length or the changes of corneal curvature, and this makes light from distant objects focus in front of the retina. That is the reason for the blurry vision when looking at far objects, unlike the clear vision when looking at close objects [1].

High myopia is a risk factor of many eye diseases, which include the cataract, myopic retinopathy, and retinal detachment [2–4]. Therefore, to find an effective method to slow the progression of myopia has become a considerable issue for the whole world. Nowadays, overnight orthokeratology is becoming more and more popular especially in the Asia-Pacific region to control the progression of myopia in young children.

Overnight orthokeratology is gas permeable rigid contact lenses that used a reverse geometry design, which is worn during sleep to reshape the front surface of the cornea for the purpose of temporary reduction of refractive errors [5]. Reduction of refractive error in myopia was thought to be the result of central corneal flattening, thickening of the midperipheral cornea, thinning of the central corneal epithelium, and peripheral vision myopic shift [6].

Analyzing wavefront aberrations resulting from orthokeratology is vital because it is the point to estimate the optical quality of the eye. A number of studies have reported that overnight orthokeratology increases ocular higher-order aberrations (HOAs) [7, 8]. In fact, recent studies have shown a consistent increase in spherical aberration and coma during overnight orthokeratology, especially coma-like aberration [9]. Another study [10] reported a sevenfold increase in spherical aberration at night 8 after the start of overnight orthokeratology. This study tried to estimate the optical quality of the eye after 6 months of orthokeratology by analyzing the changes of ocular higher-order Strehl ratio and higher-order wavefront aberrations.

## 2. Subjects and Methods

### 2.1. Subjects and Lenses

This was a prospective study of 49 eyes of 26 subjects who underwent orthokeratology (Autek China Inc.) in Boston XO material (Dk, 100 × 10-11 [cm^2^/s] [mL O^2^/mL·mmHg]). The overall diameter of the lens was 10.4–10.8 mm, and the optic zone diameter was 6.0 mm. All the subjects were required to have normal, healthy eyes. It means no history of contact lens wear, no evidence of active infection, no structural abnormalities of the eyes, and no any other ocular or systemic disease that would contraindicate lens wear. They demonstrated myopia no more than −6.00 diopters (D) and astigmatism less than or equal to 1.50 D.

In our study, all subjects have given their informed consent and the study protocol has been approved by the Institutional Review Board.

### 2.2. Measurements

Corneal topography and wavefront analysis were both performed with the OPDIII-SCAN (NIDEK, Japan). The machine can convert the corneal elevation profile into corneal wavefront data by the use of Zernike polynomials; root mean square (RMS) values were calculated for a 3.0 mm pupil. The point spread function (PSF) represents the intensity of the wavefront on the retina. The visual Strehl ratio, calculated by means of the optical transfer function method, captures the effectiveness of the retinal PSF at stimulating the neural portion of the visual system [11].

For measures of refraction and visual acuity, data were obtained by some baseline measurements before starting orthokeratology. Then, we select the lenses by averaging the data collected from the three clear photos for lens-fitting purposes. An ideal lens fit included good centration, lens movement about 1-2 mm during blinking, and no sign of lenses binding [12]. All subjects wore orthokeratology lenses while they were sleeping for at least 8 continuous hours every night. Study subjects underwent a baseline examination and followed up examinations at month 1, month 3, and month 6.

### 2.3. Data Analysis

A statistical package (SPSS 17.0) was utilized for descriptive statistics and data analysis. The repeated ANOVA was used to compare the data obtained at the latest visit with pretreatment measurements. A *P* value less than 0.05 was considered to be statistically significant.

## 3. Results

The study included 49 eyes of 26 patients (15 females and 11 males). The mean age of the patients was 10.38 ± 1.79 years (range 8 to 15 years). The mean spherical equivalent (SE) of the eyes was −3.97 ± 1.53 D. All patients returned for the 6-month follow-up visit.

### 3.1. Spherical Equivalent

The spherical equivalent was −3.97 D at the baseline. It increased 2.60 D in one month to −1.37 D. The spherical equivalent changed to −1.02 D at month 3 and −0.60 D at month 6. There were statistically significant differences between months after orthokeratology and baseline (*F* = 162.604, *P* < 0.001).

### 3.2. Corneal Surface Shape

**Figure 1:**
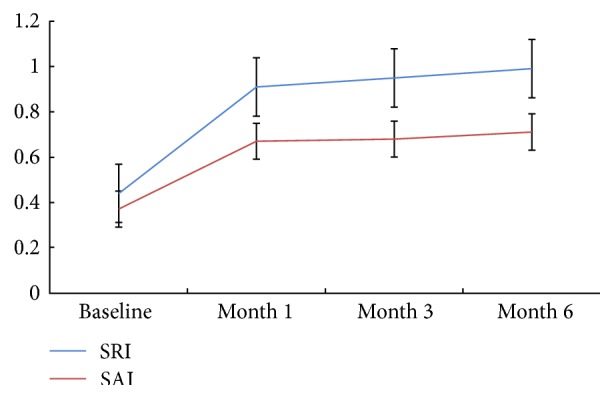
Mean values of SRI and SAI for eyes measured at baseline, month 1, month 3, and month 6 after orthokeratology.

**Figure 2:**
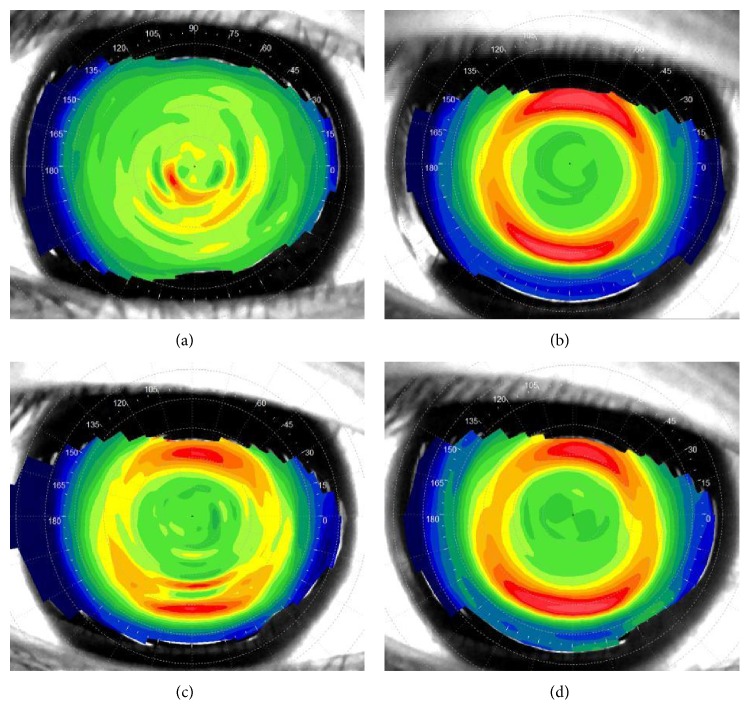
Corneal surface shape at baseline, month 1, month 3, and month 6. (a) Baseline; (b) 1 month after orthokeratology; (c) 3 months after orthokeratology; (d) 6 months after orthokeratology.

Table 1 and Figure 1 show the mean values of SRI (*F* = 103.588, *P* < 0.001) and SAI (*F* = 24.658, *P* < 0.001) for eyes measured at baseline, month 1, month 3, and month 6 after orthokeratology, and that showed a statistically significant difference during 6 months of orthokeratology through the comparison of pairwise differences between baseline and months followed.  Figure 2 show the corneal surface shape at baseline and month 1, month 3, and month 6 after orthokeratology.

### 3.3. Higher-Order Sthehl Ratio

**Figure 3:**
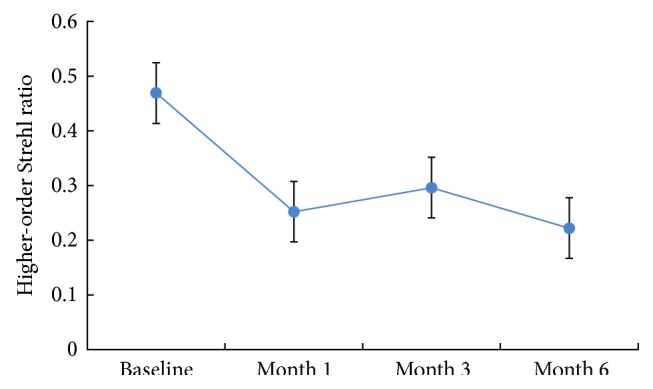
The mean of higher-order Strehl ratio for a 3.00 mm pupil diameter at baseline, month 1, month 3, and month 6 after orthokeratology.

Table 2 and Figure 3 show higher-order Strehl radio in 3.00 mm pupil diameter at baseline, 1 month, 3 months, and 6 months after orthokeratology. Higher-order Strehl radio decreased statistically significantly (*F* = 21.544, *P* < 0.001) during the follow-up period.

### 3.4. High-Order Wavefront Aberration

Table 3 summarizes the main parameters of HOA RMS. The results showed statistically significant differences in higher-order aberrations during 6 months of orthokeratology between baseline and the months (*F* = 12.018, *P* < 0.001).

**Figure 4:**
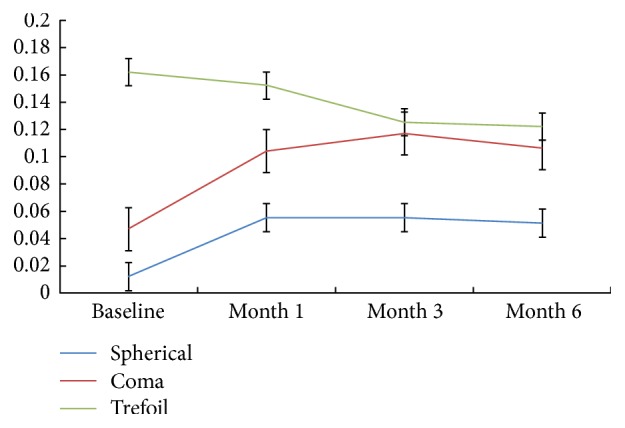
Mean values of coma, trefoil and spherical aberration at baseline, month 1, month 3, and month 6 after orthokeratology.

Table 3 and Figure 4 summarize the changes of the main parameters of HOA that showed a statistically significant difference during 6 months of orthokeratology between baseline and the months followed, except for the trefoil.

There was almost a fivefold increase in spherical aberration in 1 month. Spherical aberration showed a significant difference through the comparison of pairwise between baseline and the months followed (*F* = 11.566, *P* < 0.001). Coma aberration increased to 0.117 ± 0.145(*μ*m) at month 3 and to 0.106 ± 0.129 (*μ*m) at month 6 and showed statistically significant increases after orthokeratology (*F* = 3.536, *P* = 0.044). However, there was no difference in trefoil whenever measured within 6 months followed (*F* = 0.154, *P* = 0.722).

## 4. Discussion

We have shown that overnight orthokeratology resulted in a significant improvement in refractive error and increased corneal irregularity and ocular higher-order aberrations, especially in spherical aberration. There were plenty of reports which had similar results with us. However, as far as we know, it is the first time to estimate optical quality by using parameters which are all objective.

Overnight orthokeratology is a nonsurgical procedure to reduce refractive error and to improve the unaided vision temporarily. Plenty of studies have demonstrated that wearing long-term overnight orthokeratology can slow the progression of myopia effectively and safely [5, 13, 14]. The patients, especially young myopes with low and moderate myopia, will own useful vision during waking hours through wearing this reverse geometry-designed lenses during sleep. The reverse geometry lenses reshape the cornea intentionally by positive pressure in the center of cornea, as well as negative pressure in the midperiphery to reduce or eliminate refractive error temporarily. It makes the central cornea flattening to correct axial myopia and midperipheral cornea steepening to reduce relative peripheral hyperopia [15].

The SRI reflects the optical quality of the central cornea, and it is correlated with visual acuity, whereas the SAI reflects the asymmetry and local abnormal increases in corneal power [16]. The SRI is used to distinguish whether a reduced visual acuity results from a change in corneal topography or not, and the SAI is used as a quantitative parameter for monitoring changes in corneal topography [16].The results supported the previous reports that corneal topography is more irregular after remodeling the cornea [17] Kobayashi et al. [16] studied patients fitted with the overnight reverse geometry orthokeratology lenses and followed them up for 52 weeks and found that overnight orthokeratology not only improved uncorrected visual acuity but also increased corneal irregularity.

After wearing overnight orthokeratology, a large majority of subjects in recent studies achieved acceptable vision during daytime and reduce the need to wear spectacles [18–21] However, as with refractive surgery, visual disturbances may arise from a small or decentered central treatment zone, resulting in symptoms of ghosting and flare, particularly with a dilated pupil under low illumination conditions [10, 22]. Rah and his colleagues [23] showed that there was no difference in quality of life indices between refractive surgery and orthokeratology by using the National Eye Institute Refractive Error Quality of Life instrument (NEI-RQL 42). There are also a lot of studies hold that the overnight orthokeratology can induce significant changes in optical quality [8, 24, 25].

The Strehl ratio reflects the level of image quality in the presence of wavefront aberrations. It is one of the most objective parameters which are related to optical quality [11]. It is defined as the ratio of the peak diffraction intensities of a measured eye to those of an aberration-free eye [26]. The value of the Strehl ratio is generally between 0 and 1. For an optical system, if the value is higher than 0.8, it can be expected that this optical system is perfect [27]. In our study, the higher-order Strehl ratio for 3 mm pupil diameters decreased significantly after remodeling the cornea for 1 month.

Analyzing higher-order aberrations resulting from orthokeratology is important because it represents the effect of corneal remodeling on the optical quality of the entire eye after orthokeratology. A number of studies have reported that overnight orthokeratology increases the RMS of higher-order aberrations, especially spherical aberration and coma aberration [8, 28, 29]. In orthokeratology, the increase in spherical aberration is mainly attributed to the nonphysiological oblate cornea after treatment, and the increase in coma aberration reflects contact lens decentration [30]. Stillitano et al. [10] have reported that trefoil increased significantly statistically after overnight orthokeratology, while there is no significant difference in trefoil in our study.

Our study had some limitations. First, month 1 is the first point we evaluated corneal surface shape and optical quality after orthokeratology. We are interested to know how the parameters change after orthokeratology within one month. Second, there were 26 subjects (49 eyes) in our study, and the samples and the parameters are limited. We will expand the number of samples and explore other parameters to find out how the parameters are changing in the future.

## Figures and Tables

**Table 1:** Corneal reshaping parameters (mean ± SD) at baseline, month 1, month 3, and month 6 after orthokeratology.

	Baseline	Month 1	Month 3	Month 6	*F*	*P*
SRI	0.44 ± 0.18	0.91 ± 0.26	0.95 ± 0.30	0.99 ± 0.25	103.588	<0.001
SAI	0.37 ± 0.12	0.67 ± 0.39	0.68 ± 0.34	0.71 ± 0.36	24.658	<0.001

**Table 2:** Mean ± SD Strehl ratio of higher-order for a 3.00 mm pupil diameter at baseline, month 1, month 3, and month 6 after orthokeratology.

	Baseline	Month 1	Month 3	Month 6	*F*	*P*
Strehl ratio	0.469 ± 0.234	0.252 ± 0.171	0.296 ± 0.202	0.222 ± 0.151	21.544	<0.001

**Table 3:** Mean ± SD root mean square values (*μ*m) of higher-order aberrations for a 3.00 mm pupil diameter at baseline, month 1, month 3, and month 6 after orthokeratology.

	Baseline	Month 1	Month 3	Month 6	*F*	*P*
HOA RMS	0.109 ± 0.079	0.209 ± 0.215	0.213 ± 0.183	0.215 ± 0.178	12.018	<0.001
Spherical	0.012 ± 0.030	0.055 ± 0.071	0.055 ± 0.065	0.051 ± 0.034	11.566	<0.001
Coma	0.047 ± 0.147	0.104 ± 0.144	0.117 ± 0.145	0.106 ± 0.129	3.536	0.044
Trefoil	0.162 ± 0.659	0.152 ± 0.198	0.125 ± 0.115	0.122 ± 0.114	0.154	0.722
